# Fucoidan-Based Combination Strategies in Cancer Therapy: Biological Basis, Current Evidence, and Future Perspectives

**DOI:** 10.3390/ijms27167370

**Published:** 2026-08-18

**Authors:** Yumi Jang

**Affiliations:** 1 Department of Food Science and Nutrition, University of Ulsan, Ulsan 44610, Republic of Korea; yjang@ulsan.ac.kr; Tel.: +82-52-259-2374; 2 Basic-Clinical Convergence Research Institute, University of Ulsan, Ulsan 44610, Republic of Korea

**Keywords:** fucoidan, combination therapy, cancer, marine-derived polysaccharide, chemotherapy, natural bioactive compounds

## Abstract

Combination therapy has emerged as an effective strategy to enhance anticancer efficacy while overcoming the limitations of conventional cancer treatment, including systemic toxicity and therapeutic resistance. Among marine-derived bioactive compounds, fucoidan, a sulfated polysaccharide isolated from brown seaweeds, has attracted considerable attention because of its multitarget biological activities, favorable safety profile, and ability to enhance therapeutic responses. Increasing evidence suggests that fucoidan may be particularly valuable as a multifunctional combination partner rather than as a standalone anticancer agent, capable of potentiating the efficacy of conventional chemotherapy, targeted therapy, and, to a much lesser extent, natural bioactive compounds. In this review, we summarize the biological characteristics that make fucoidan an attractive combination partner and provide a comprehensive overview of current evidence regarding fucoidan-based combination strategies for cancer therapy. We further discuss the molecular mechanisms underlying these combination effects, including the regulation of apoptosis, autophagy, tumor microenvironment remodeling, and oncogenic signaling pathways associated with therapeutic resistance. Although combinations with conventional anticancer therapies have been extensively investigated, studies involving natural bioactive compounds remain remarkably limited, highlighting an important opportunity for future research. Collectively, current evidence, derived largely from preclinical studies, supports the potential of fucoidan as a promising multifunctional adjuvant for cancer combination therapy, while further standardization, mechanistic studies, and clinical validation will be essential for its successful translation into clinical practice.

## 1. Introduction

Cancer remains one of the leading causes of morbidity and mortality worldwide despite remarkable advances in cancer diagnosis and therapeutic interventions. According to the Global Cancer Observatory (GLOBOCAN), both the incidence and mortality of cancer continue to increase owing to population aging, environmental exposure, and lifestyle changes [1]. Although conventional therapeutic modalities, including surgery, chemotherapy, radiotherapy, targeted therapy, and immunotherapy, have substantially improved patient survival, their clinical efficacy is frequently limited by systemic toxicity, intrinsic or acquired drug resistance, tumor recurrence, and metastatic progression. These challenges have driven increasing efforts to develop safer and more effective therapeutic strategies capable of enhancing therapeutic efficacy while minimizing adverse effects [2].

Natural products have long been recognized as an important source of anticancer agents because of their structural diversity and wide range of biological activities. Unlike conventional chemotherapeutic drugs that primarily target specific molecular pathways, many natural bioactive compounds often influence multiple biological processes by modulating diverse signaling pathways involved in cancer initiation and progression. Consequently, increasing attention has been directed toward their use not only as direct anticancer agents but also as adjuvants that enhance the efficacy of existing cancer therapies through combination strategies [3,4].

For several decades, research on natural product-based cancer therapy has been dominated by plant-derived phytochemicals. Representative compounds such as curcumin, resveratrol, quercetin, and epigallocatechin-3-gallate (EGCG) have demonstrated promising anticancer activities by modulating apoptosis, autophagy, oxidative stress, inflammation, angiogenesis, metastasis, and multiple oncogenic signaling pathways [5]. More importantly, numerous studies have shown that these phytochemicals exhibit synergistic anticancer effects when combined with conventional chemotherapeutic agents or other natural compounds [6,7,8]. Such combination strategies simultaneously target multiple oncogenic pathways, improve therapeutic efficacy, reduce effective drug dosage, alleviate treatment-associated toxicity, and overcome drug resistance. Consequently, combination therapy using natural bioactive compounds has emerged as an attractive strategy for improving cancer treatment [3,4].

Compared with plant-derived phytochemicals, marine-derived natural products have historically received relatively less attention despite the extraordinary biodiversity and unique chemical structures found in marine organisms [9,10]. However, recent advances in marine biotechnology, natural product chemistry, and analytical techniques have accelerated the discovery and development of marine-derived compounds with considerable therapeutic potential [4,10]. Marine organisms produce structurally diverse bioactive compounds, including polysaccharides, peptides, carotenoids, alkaloids, terpenoids, and polyphenols, many of which exhibit antioxidant, anti-inflammatory, immunomodulatory, antiviral, and anticancer activities [9,10]. Consequently, marine-derived natural products have emerged as an important source of novel therapeutic agents for cancer and other chronic diseases [4,10,11].

Among marine-derived bioactive compounds, fucoidan, a sulfated polysaccharide predominantly isolated from brown seaweeds such as *Fucus vesiculosus*, *Undaria pinnatifida*, and *Laminaria japonica*, has emerged as one of the most extensively investigated marine natural products because of its diverse biological activities, including antioxidant, anti-inflammatory, immunomodulatory, and anticancer effects [12,13,14]. Numerous experimental studies have demonstrated that fucoidan exhibits intrinsic anticancer activity in various cancer models while showing relatively low cytotoxicity toward normal cells [15,16,17,18,19]. Although fucoidan has shown promising anticancer efficacy as a single agent, accumulating evidence suggests that its therapeutic potential may be further enhanced when combined with conventional anticancer therapies or other natural bioactive compounds [13,14,20].

Indeed, accumulating evidence indicates that fucoidan enhances the efficacy of conventional anticancer therapies, including 5-fluorouracil, cisplatin, oxaliplatin, doxorubicin, paclitaxel, molecular targeted therapies, and immunotherapeutic approaches [21,22,23,24]. In addition, several studies have investigated combinations of fucoidan with natural bioactive compounds, including resveratrol and vitamin C, and have reported enhanced anticancer effects [25,26]. Although evidence supporting these combinations remains limited, the available findings suggest that combining marine-derived fucoidan with complementary natural bioactive compounds represents a promising and largely underexplored strategy for cancer therapy. Collectively, these studies indicate that fucoidan should be regarded not only as an independent anticancer agent but also as a multifunctional partner capable of enhancing the therapeutic efficacy of diverse anticancer interventions.

While numerous reviews have comprehensively summarized the structural characteristics, extraction methods, biological activities, and pharmacological properties of fucoidan, relatively few have specifically focused on its role as a multifunctional partner in combination therapy [27]. Moreover, the available evidence remains scattered across different therapeutic partners, cancer types, and molecular mechanisms, making it difficult to obtain an integrated understanding of this rapidly evolving field. In particular, combinations of fucoidan with natural bioactive compounds have received relatively little systematic attention despite their considerable therapeutic potential. Therefore, a comprehensive review focusing on fucoidan-based combination therapy is both timely and warranted. In this review, we summarize the biological characteristics supporting fucoidan-based combination therapy, discuss current evidence regarding the combined use of fucoidan with conventional anticancer therapies and natural bioactive compounds, highlight the molecular mechanisms underlying these combination effects, and address current challenges and future perspectives for the clinical translation of fucoidan-based combination strategies [14,27].

## 2. Biological Characteristics of Fucoidan Supporting Combination Therapy

**Figure 1:**
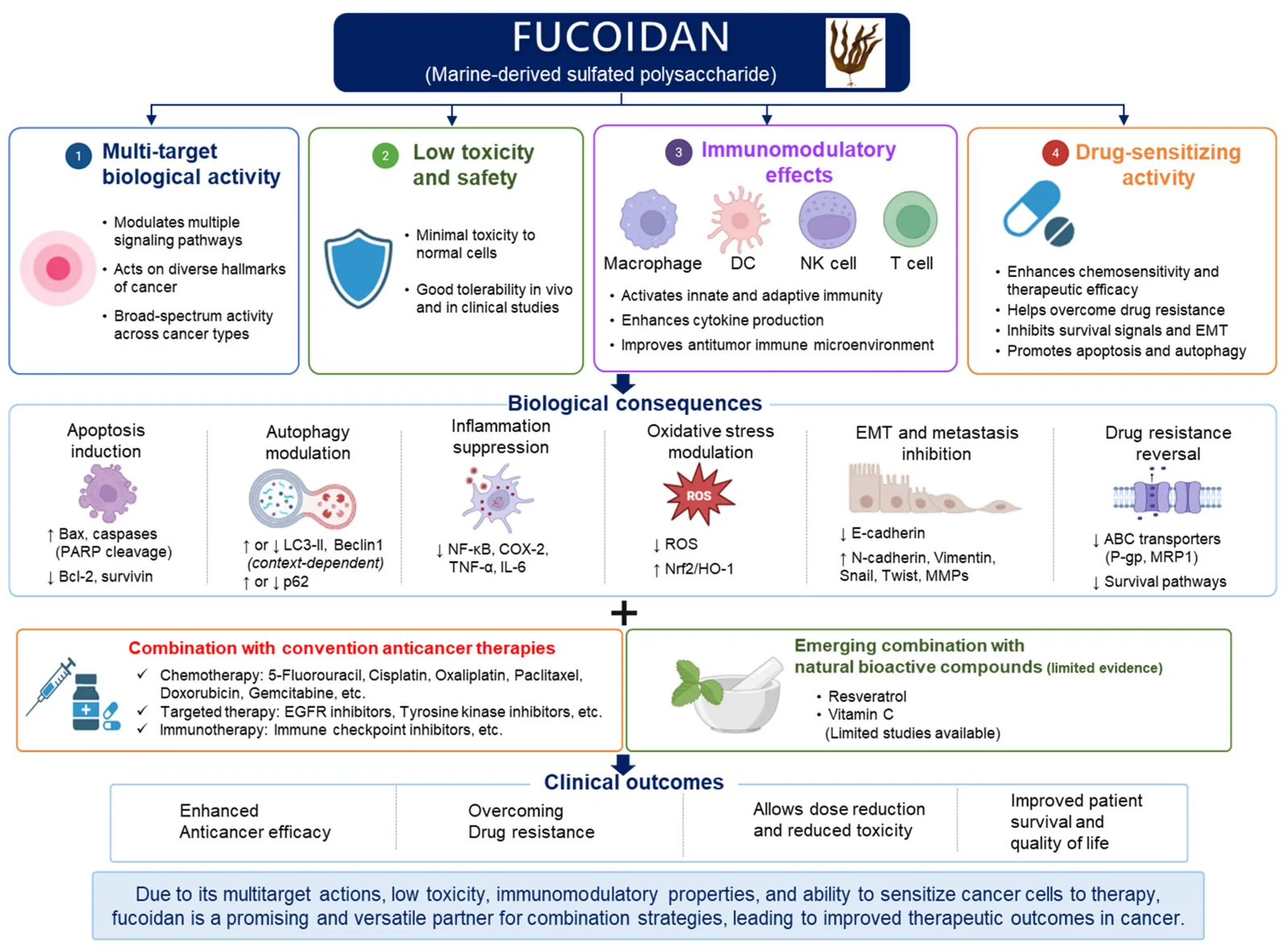
Biological basis of fucoidan-based combination therapy in cancer. Fucoidan possesses multiple biological properties, including multitarget biological activity, favorable safety, immunomodulatory effects, and drug-sensitizing activity, which contribute to enhanced therapeutic efficacy in combination with conventional anticancer therapies and natural bioactive compounds. ABC, ATP-binding cassette; DC, dendritic cell; EMT, epithelial–mesenchymal transition; HO-1, heme oxygenase-1; IL-6, interleukin-6; MMPs, matrix metalloproteinases; MRP1, multidrug resistance-associated protein 1; NF-κB, nuclear factor kappa B; NK, natural killer; Nrf2, nuclear factor erythroid 2-related factor 2; PARP, poly(ADP-ribose) polymerase; P-gp, P-glycoprotein; ROS, reactive oxygen species; TNF-α, tumor necrosis factor-α; ↑, increased; ↓, decreased. Created in BioRender. Jang, Y. (2026) https://BioRender.com/y6at9o7.

Fucoidan has emerged as one of the most extensively investigated marine-derived polysaccharides owing to its broad pharmacological activities, favorable safety profile, and diverse biological properties [13,14,28]. Unlike many conventional anticancer agents that primarily target a limited number of molecular pathways, fucoidan exerts pleiotropic biological effects through the coordinated regulation of multiple cancer-associated signaling networks involved in tumor growth, survival, metastasis, inflammation, and immune regulation [13,29]. Although numerous experimental studies have demonstrated the intrinsic anticancer activity of fucoidan as a single agent in various cancer models [15,16,17,18,19,30,31], accumulating evidence indicates that its greatest therapeutic value lies in combination therapy, where it enhances the efficacy of conventional anticancer drugs and natural bioactive compounds while potentially reducing treatment-associated toxicity [13,14]. Several biological characteristics contribute to the potential of fucoidan as a combination partner for cancer therapy, including multitarget biological activity, low toxicity, immunomodulatory capacity, drug-sensitizing properties, and advantageous physicochemical characteristics [13,14,28] (Figure 1).

### 2.1. Multi-Target Biological Activity

One of the major advantages of fucoidan is its ability to simultaneously regulate multiple biological processes associated with tumor development and progression [13,14,29]. Because cancer is driven by complex and interconnected signaling networks, therapeutic strategies targeting a single pathway frequently result in compensatory pathway activation and therapeutic resistance [2]. In contrast, fucoidan modulates numerous cancer-associated signaling pathways, including PI3K/Akt/mTOR, MAPK, NF-κB, STAT3, Wnt/β-catenin, and TGF-β signaling, thereby influencing apoptosis, autophagy, inflammation, angiogenesis, epithelial–mesenchymal transition (EMT), migration, and invasion [13,28,29,32]. Experimental studies have demonstrated that these multitarget effects are associated with activation of apoptotic signaling, induction of autophagy, inhibition of angiogenesis, and suppression of tumor cell migration and invasion through the modulation of multiple intracellular signaling pathways [16,17,18,19,30]. Through these complementary biological activities, fucoidan provides a strong mechanistic basis for enhancing the efficacy of combination therapy with conventional anticancer agents and natural bioactive compounds [13,14,20].

### 2.2. Favorable Safety Profile

An ideal combination partner should enhance therapeutic efficacy without substantially increasing treatment-related toxicity. Compared with conventional cytotoxic drugs, fucoidan exhibits remarkably low toxicity toward normal cells while maintaining selective anticancer activity across a wide range of experimental models [13,14,28].

Preclinical evidence from in vitro and animal studies generally supports a favorable safety profile of fucoidan, with relatively low toxicity toward normal cells and tissues [13,14,28]. Available human studies, although limited in number and scope, also suggest that fucoidan is generally well tolerated when used as a dietary supplement or adjunct to conventional therapy [33,34,35,36]. However, these clinical findings remain limited and are not sufficient to establish the long-term safety or therapeutic efficacy of fucoidan in cancer patients. Recent reviews similarly conclude that the available clinical evidence is encouraging but remains insufficient, emphasizing the need for well-designed clinical trials [35,36]. These characteristics support the potential use of fucoidan in combination therapy, where minimizing cumulative toxicity is important for maintaining treatment intensity and patients’ quality of life [13,28].

### 2.3. Drug-Sensitizing Properties

Increasing evidence indicates that fucoidan functions not only as an anticancer agent but also as a therapeutic sensitizer that enhances the efficacy of existing anticancer therapies through complementary molecular mechanisms rather than replacing them [13,14,29]. This sensitizing effect is largely attributed to the ability of fucoidan to modulate multiple pathways involved in apoptosis, autophagy, oxidative stress, inflammation, angiogenesis, and the tumor microenvironment, all of which contribute to treatment response and drug resistance [13,29]. Representative studies have demonstrated that fucoidan enhances the efficacy of 5-fluorouracil, cisplatin, doxorubicin, paclitaxel, oxaliplatin, sorafenib, and bevacizumab, resulting in enhanced apoptosis, improved chemosensitivity, suppression of angiogenic signaling, and modulation of the tumor microenvironment. Collectively, these findings support the application of fucoidan as a multifunctional adjuvant capable of improving therapeutic efficacy while potentially overcoming therapeutic resistance, thereby providing a strong rationale for combination-based cancer therapy [13,14,21,22,23,24,37].

### 2.4. Immunomodulatory Activity

In addition to its direct anticancer effects, fucoidan possesses significant immunomodulatory activity. Previous studies have demonstrated that fucoidan regulates both innate and adaptive immune responses by modulating macrophages, dendritic cells, natural killer (NK) cells, and T lymphocytes [14,29]. Fucoidan also influences macrophage polarization, cytokine production, and antitumor immune surveillance, thereby contributing to a more favorable tumor immune microenvironment [24,29]. These immunomodulatory properties further support the use of fucoidan as a promising partner for combination therapy, particularly in conjunction with targeted therapies and immunotherapy [14,24].

### 2.5. Favorable Physicochemical Properties

Besides its biological activities, fucoidan possesses physicochemical properties that further support its application in combination therapy. As a naturally occurring sulfated polysaccharide, fucoidan is water-soluble, biocompatible, biodegradable, and readily incorporated into various drug delivery systems. These characteristics have facilitated the development of fucoidan-based nanoparticles, liposomes, hydrogels, and other delivery platforms designed to improve drug stability, tumor targeting, and controlled drug release. In addition to serving as a bioactive therapeutic molecule, fucoidan can function as a multifunctional carrier that enhances the delivery and therapeutic performance of co-administered anticancer agents, further expanding its potential in combination-based cancer therapy [27,32].

Collectively, these unique properties provide a strong rationale for the application of fucoidan as a multifunctional partner in combination-based cancer therapy. Representative studies demonstrating these combination strategies are discussed in the following sections.

## 3. Fucoidan-Based Combination Strategies in Cancer Therapy

Although fucoidan has demonstrated anticancer activity in a variety of experimental models, recent studies have increasingly focused on its application in combination therapy. Owing to its diverse biological activities and favorable physicochemical properties, fucoidan has been investigated in combination with conventional anticancer therapies, such as chemotherapy, targeted therapy, and immunotherapy, as well as with natural bioactive compounds. In most experimental models, these combinations produced greater anticancer effects than either treatment alone by promoting apoptosis, modulating autophagy, regulating antitumor immunity, and inhibiting angiogenic signaling. Representative studies are summarized in Table 1 [14,29].

### 3.1. Combination with Conventional Anticancer Therapies

To date, most fucoidan-based combination studies have focused on conventional anticancer therapies, particularly chemotherapy. Importantly, the evidence supporting these combinations is derived predominantly from preclinical studies, including in vitro cancer cell models and in vivo animal models, whereas clinical evidence specifically evaluating the efficacy of fucoidan-based combination therapy remains limited. Various classes of anticancer agents, including fluoropyrimidines, platinum-based drugs, taxanes, anthracyclines, targeted therapies, and more recently immunotherapeutic agents, have been examined in combination with fucoidan. Among these, the combination with 5-fluorouracil has been one of the most extensively investigated in colorectal cancer models. Huang et al. demonstrated that low-molecular-weight fucoidan significantly enhanced the antiproliferative activity of 5-fluorouracil by suppressing c-MET/KRAS/ERK and PI3K/AKT signaling, resulting in increased apoptosis and reduced cancer cell migration [22].

Subsequent studies expanded these findings to other chemotherapeutic agents. Fucoidan significantly potentiated the anticancer activities of cisplatin, doxorubicin, and paclitaxel in breast cancer cells by inducing cell-cycle arrest and caspase-dependent apoptosis while reducing the toxicity of cisplatin and doxorubicin toward non-malignant breast epithelial cells, suggesting an improved therapeutic index [21]. More recently, Zhang et al. reported that fucoidan enhanced the sensitivity of breast cancer cells to cisplatin and doxorubicin through activation of autophagy, characterized by increased LC3B expression and decreased p62 levels [37]. In pancreatic ductal adenocarcinoma, low-molecular-weight fucoidan also enhanced the efficacy of oxaliplatin by promoting TLR4/NF-κB-mediated M1 macrophage polarization and remodeling the immunosuppressive tumor microenvironment [24].

Beyond conventional chemotherapy, fucoidan has also demonstrated promising potential in combination with targeted therapies. In hepatocellular carcinoma models, fucoidan synergistically enhanced the antitumor effects of sorafenib and bevacizumab, resulting in reduced tumor cell viability and migration, suppression of VEGF/PI3K/AKT/mTOR and KRAS/BRAF/MAPK signaling, and increased caspase-dependent apoptosis in both in vitro and in vivo models [23]. However, enhanced effects are not consistently observed across cancer cell types. Oh et al. reported that the combination of fucoidan with the EGFR/HER2 inhibitor lapatinib produced cell type-dependent responses, with a tendency toward enhanced effects in some cancer cells but reduced effects in others, emphasizing the importance of tumor-specific evaluation when designing fucoidan-based combination therapies [38].

Fucoidan has also shown potential as an adjunct to immune checkpoint blockade in preclinical models. Yang et al. reported that a fucoidan-supplemented diet enhanced the antitumor activity of anti-PD-1 treatment by promoting tumor-infiltrating CD8+ T-cell activation, with JAK/STAT signaling involved in the activation of T cells [39]. Li et al. further showed that fucoidan enhanced the response to anti-PD-1 treatment in a breast cancer model, accompanied by modulation of the gut microbiota, enhanced effector T-cell function, and reduced Treg cell levels [40]. More recently, fucoidan derived from Durvillaea antarctica enhanced the antitumor effect of anti-PD-L1 treatment in a CT-26 tumor model through TLR4-dependent activation of dendritic cells and T cells [41]. Together, these preclinical findings suggest that the immunomodulatory properties of fucoidan may contribute to improved responses to immune checkpoint blockade, although clinical validation is still required.

Taken together, these preclinical findings suggest that fucoidan may enhance the efficacy of anticancer therapies through multiple complementary mechanisms rather than acting through a single molecular target, supporting further investigation of fucoidan as a therapeutic sensitizer [22,29,42].

### 3.2. Combination with Natural Bioactive Compounds

Compared with the rapidly expanding literature on fucoidan combined with conventional anticancer therapies, studies investigating combinations of fucoidan with natural bioactive compounds in cancer remain limited. Nevertheless, the available evidence suggests that combining fucoidan with naturally occurring bioactive compounds with complementary biological activities may represent a promising strategy for improving therapeutic efficacy.

One of the earliest studies demonstrated that sulfated α-L-fucan isolated from Saccharina cichorioides significantly enhanced resveratrol-induced apoptosis in HCT116 colon carcinoma cells through activation of caspase-dependent apoptotic pathways and increased PARP cleavage, thereby producing greater inhibition of cancer cell survival than resveratrol alone [25]. More recently, fucoidan combined with vitamin C synergistically induced apoptosis and suppressed colon cancer cell growth, further supporting the feasibility of combining marine-derived polysaccharides with naturally occurring bioactive compounds for cancer therapy [26].

Although direct evidence in cancer remains limited, potential interactions between fucoidan and natural bioactive compounds have also been reported in non-cancer disease models. For example, the combination of fucoidan and chito-oligosaccharides exhibited greater anti-inflammatory and intestinal barrier-protective effects than either compound alone in a DSS-induced colitis model through complementary regulation of inflammatory mediators and epithelial barrier function [43]. However, this evidence was obtained from a non-cancer model and therefore cannot be directly extrapolated to cancer therapy. Given the pivotal roles of oxidative stress, chronic inflammation, and immune dysregulation in tumor initiation and progression, these findings may nevertheless provide a basis for future investigation of fucoidan-based combinations with natural bioactive compounds in cancer.

Overall, current evidence supports further investigation of fucoidan as a combination partner for both conventional anticancer therapies and natural bioactive compounds. However, unlike the extensive body of evidence supporting combinations with conventional anticancer therapies, studies involving natural bioactive compounds remain limited, highlighting an important research gap [14,29]. Future studies should systematically evaluate combinations of fucoidan with diverse dietary bioactive compounds, including polyphenols, flavonoids, vitamins, peptides, and other marine- or plant-derived molecules, to determine whether they enhance therapeutic effects and to better understand the underlying molecular mechanisms in cancer [14,29]. The molecular mechanisms underlying these combination strategies are discussed in the following section.

## 4. Molecular Mechanisms Underlying the Effects of Fucoidan-Based Combination Therapy

**Figure 2:**
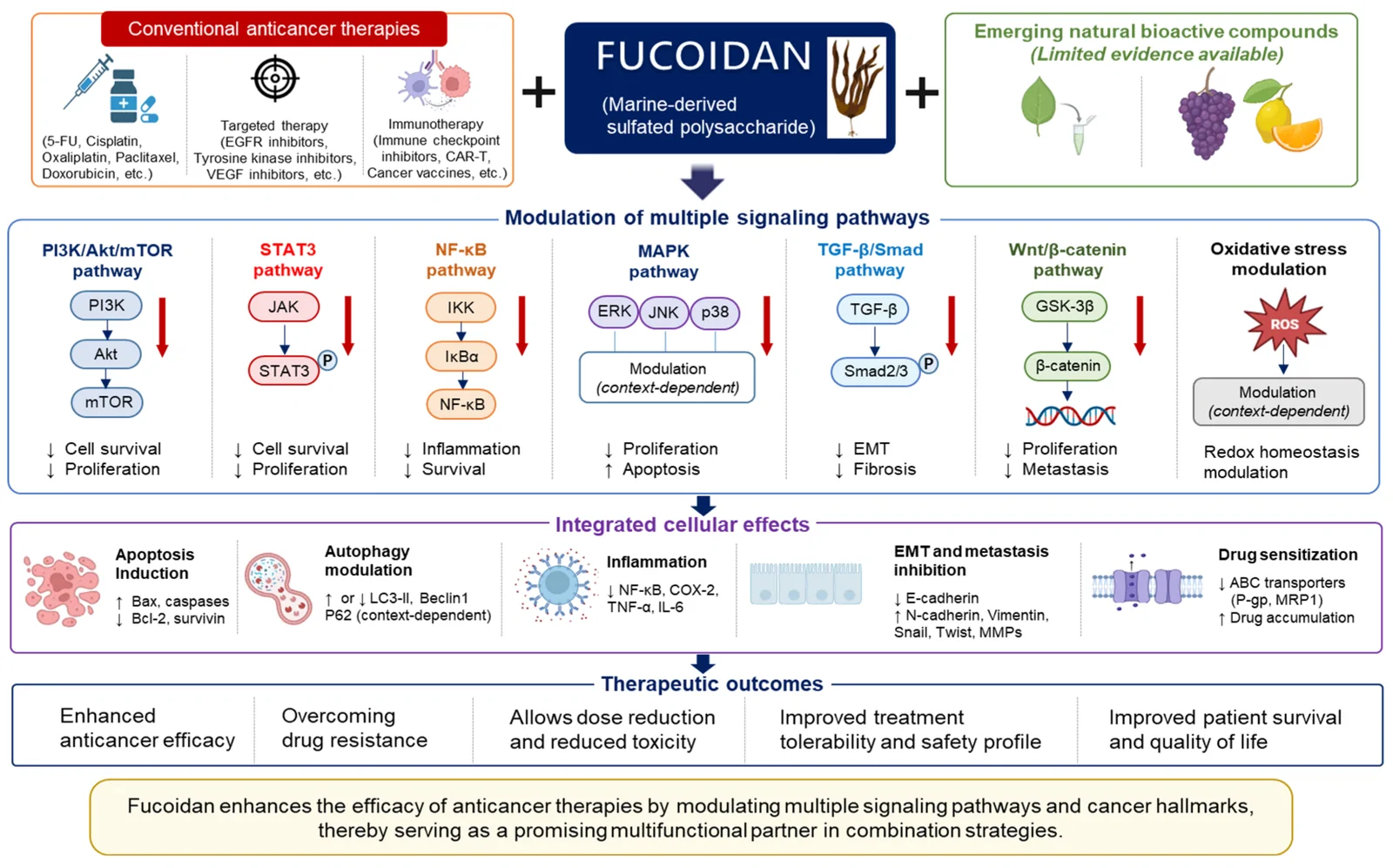
Molecular mechanisms underlying fucoidan-based combination therapy. Fucoidan enhances the efficacy of combination therapy by modulating multiple signaling pathways involved in apoptosis, autophagy, inflammation, oxidative stress, EMT, and therapeutic resistance, leading to improved anticancer efficacy and reduced toxicity. 5-FU, 5-fluorouracil; CAR-T, chimeric antigen receptor T cell; EGFR, epidermal growth factor receptor; EMT, epithelial–mesenchymal transition; HER2, human epidermal growth factor receptor 2; MAPK, mitogen-activated protein kinase; MMPs, matrix metalloproteinases; MRP1, multidrug resistance-associated protein 1; NF-κB, nuclear factor kappa B; PI3K, phosphoinositide 3-kinase; ROS, reactive oxygen species; STAT3, signal transducer and activator of transcription 3; TGF-β, transforming growth factor-β; TNF-α, tumor necrosis factor-α; VEGF, vascular endothelial growth factor; ↑, increased; ↓, decreased. Created in BioRender. Jang, Y. (2026) https://BioRender.com/y6at9o7.

The enhanced anticancer efficacy of fucoidan-based combination therapy is attributed to the coordinated modulation of multiple molecular pathways rather than the regulation of a single target. Although the underlying mechanisms vary depending on the cancer type and therapeutic partner, accumulating evidence indicates that fucoidan enhances therapeutic responses through the regulation of apoptosis, autophagy, oxidative stress, the tumor microenvironment, and multiple oncogenic signaling pathways associated with therapeutic resistance. Together, these complementary mechanisms provide the molecular basis for the enhanced therapeutic efficacy observed in combination with conventional anticancer therapies and natural bioactive compounds. The major molecular mechanisms involved in these combination strategies are summarized in Figure 2 and discussed in the following sections [13,14,29].

### 4.1. Promotion of Apoptosis

Apoptosis is the most consistently reported mechanism underlying the synergistic anticancer effects of fucoidan-based combination therapy. Numerous studies have demonstrated that fucoidan enhances apoptosis when combined with chemotherapeutic agents or natural bioactive compounds. This enhanced apoptotic response is characterized by activation of caspase-3, caspase-7, and caspase-9, an increased Bax/Bcl-2 ratio, PARP cleavage, and accumulation of cells in the sub-G1 phase [22,32].

For example, fucoidan significantly potentiated the cytotoxic effects of cisplatin, doxorubicin, and paclitaxel in MCF-7 breast cancer cells by inducing G2/M cell-cycle arrest and activating caspase-dependent apoptosis while exerting minimal toxicity toward normal breast epithelial cells [21]. Likewise, sulfated α-L-fucan isolated from Saccharina cichorioides markedly enhanced resveratrol-induced apoptosis through increased caspase activation and PARP cleavage in HCT116 colon cancer cells [25]. Similar enhancement of apoptotic signaling has been reported in combination with 5-fluorouracil, sorafenib, and vitamin C, supporting apoptosis as a common downstream mechanism underlying the synergistic effects of fucoidan-based combination therapy [22,23,26,32].

### 4.2. Modulation of Autophagy

Autophagy is another important mechanism involved in fucoidan-based combination therapy. In cancer cells, autophagy can have opposing functions depending on the cellular context. Under metabolic or therapeutic stress, autophagy may promote cell survival by maintaining cellular homeostasis, whereas sustained autophagic activation may also be associated with cell death or increased sensitivity to anticancer treatment [32,44].

In studies of fucoidan, autophagy has been associated with growth inhibition or enhanced therapeutic response. Zhang et al. demonstrated that fucoidan significantly increased the sensitivity of breast cancer cells to cisplatin and doxorubicin, accompanied by increased LC3B expression and decreased p62 levels [37]. Park et al. also reported that fucoidan induced autophagy together with apoptosis in AGS gastric cancer cells, as indicated by autophagosome formation, LC3-I to LC3-II conversion, and increased Beclin-1 expression [45]. However, the direct contribution of autophagy to fucoidan-induced cell death was not established in this study. These findings indicate that the role of autophagy may depend on the cancer type, treatment conditions, and extent of autophagic activation, and further studies are needed to distinguish cytoprotective from cell death-associated autophagy.

### 4.3. Remodeling of the Tumor Microenvironment

Beyond its direct cytotoxic effects on cancer cells, fucoidan enhances therapeutic efficacy by modulating antitumor immunity and angiogenesis, thereby remodeling the tumor microenvironment [32,36]. These changes contribute to a tumor microenvironment that is less favorable for tumor progression and more responsive to anticancer therapy.

For example, Deng et al. demonstrated that low-molecular-weight fucoidan enhanced the antitumor activity of oxaliplatin in pancreatic ductal adenocarcinoma by activating the TLR4/NF-κB pathway and promoting macrophage polarization toward the tumoricidal M1 phenotype. This resulted in increased M1 macrophage infiltration, reversal of the immunosuppressive tumor microenvironment, and improved therapeutic efficacy [24].

Fucoidan also enhances antiangiogenic therapy. In hepatocellular carcinoma models, combination treatment with fucoidan and sorafenib or bevacizumab suppressed VEGF/PI3K/Akt/mTOR and KRAS/BRAF/MAPK signaling, reduced angiogenesis and tumor cell migration, and enhanced apoptosis in both in vitro and in vivo models [23]. Collectively, these findings highlight the importance of tumor microenvironment remodeling in the enhanced therapeutic efficacy of fucoidan-based combination therapy [32,36].

### 4.4. Modulation of Oncogenic Signaling Pathways and Drug Resistance

Resistance to anticancer therapy remains a major challenge in cancer treatment. Increasing evidence suggests that fucoidan enhances therapeutic responsiveness through coordinated modulation of multiple oncogenic signaling pathways associated with tumor progression and drug resistance [32,36].

Among these pathways, PI3K/Akt, MAPK, MET/KRAS/ERK, STAT3, and epithelial–mesenchymal transition (EMT)-related signaling have been identified as key molecular targets of fucoidan [32,36]. Huang et al. demonstrated that low-molecular-weight fucoidan significantly enhanced the efficacy of 5-fluorouracil by suppressing MET/KRAS/ERK and PI3K/Akt signaling, thereby reducing cell proliferation and migration while enhancing apoptosis in colorectal cancer cells [22]. Similar modulation of PI3K/Akt/mTOR and MAPK signaling was observed following combination treatment with fucoidan and sorafenib or bevacizumab in hepatocellular carcinoma models [23]. In addition, fucoidan has been reported to modulate EMT-related markers and transcription factors in breast cancer models, increasing epithelial marker expression while reducing mesenchymal marker expression and the EMT-associated transcriptional repressors Snail, Slug, and Twist [32]. These changes were associated with reduced tumor growth and metastatic potential [32]. Collectively, these findings suggest that fucoidan improves therapeutic responsiveness through coordinated regulation of multiple oncogenic signaling networks rather than by targeting a single resistance mechanism [32,36].

### 4.5. Summary

Current evidence indicates that the enhanced effects of fucoidan-based combination therapy result from the coordinated regulation of multiple molecular pathways rather than a single target. By simultaneously promoting apoptosis, modulating autophagy, remodeling the tumor microenvironment, and regulating oncogenic signaling pathways associated with therapeutic resistance, fucoidan enhances the efficacy of conventional chemotherapeutic agents, targeted therapies, and natural bioactive compounds. Collectively, these findings highlight the therapeutic potential of fucoidan as a versatile combination partner and provide a molecular basis for further translational and clinical studies aimed at improving cancer treatment outcomes [29,32,36].

## 5. Challenges and Future Perspectives

Despite the encouraging preclinical evidence supporting fucoidan-based combination therapy, several important challenges must be addressed before its clinical translation can be fully realized. One of the major limitations is the structural heterogeneity of fucoidan. Its molecular weight, monosaccharide composition, degree of sulfation, and branching structure vary considerably depending on algal species, harvesting season, extraction procedures, and purification methods. These structural variations substantially influence its biological activity, making direct comparisons among studies difficult and limiting experimental reproducibility. Therefore, the establishment of standardized extraction protocols and well-characterized fucoidan preparations will be essential for future mechanistic investigations and clinical applications [13,14].

Another important challenge is the limited understanding of the pharmacokinetic characteristics of fucoidan. Although numerous studies have demonstrated favorable safety profiles and relatively low toxicity, its oral bioavailability, absorption, tissue distribution, metabolism, and elimination remain poorly understood. Moreover, most current evidence comes from in vitro studies and small animal models, while well-designed clinical trials evaluating fucoidan as an adjuvant to standard anticancer therapies remain limited. Nevertheless, clinical studies have reported potential benefits of fucoidan supplementation during conventional anticancer therapy. In patients with colorectal cancer, fucoidan supplementation has been associated with improved disease control and reduced chemotherapy-related fatigue, although clear survival benefits have not been established [33,46]. More recently, a randomized, double-blind, placebo-controlled trial in patients with unresectable hepatocellular carcinoma demonstrated that low-molecular-weight fucoidan combined with transarterial chemoembolization improved disease control and preserved liver function without increasing severe adverse events [47]. In patients with locally advanced rectal cancer receiving neoadjuvant concurrent chemoradiotherapy, low-molecular-weight fucoidan improved quality of life and reduced treatment-related fatigue and skin symptoms, although disease-free and overall survival were not significantly improved [48]. These recent findings provide encouraging clinical evidence for fucoidan as an adjunct to conventional cancer therapy; however, larger clinical trials are needed to establish its therapeutic efficacy and survival benefits. Consequently, the optimal dosage, treatment schedule, and clinical efficacy of fucoidan-based combination therapy have yet to be established [13,14].

From the perspective of combination therapy, future studies should move beyond demonstrating enhanced anticancer activity and rigorously evaluate synergistic interactions using quantitative approaches, including combination index (CI) analysis and isobologram analysis, together with mechanistic validation using genetic or pharmacological interventions. Importantly, while combinations of fucoidan with conventional chemotherapeutic agents have been extensively investigated, studies involving natural bioactive compounds remain limited. Expanding these investigations to include diverse dietary phytochemicals, flavonoids, vitamins, peptides, and other marine- or plant-derived bioactive compounds may facilitate the development of safer multitarget therapeutic strategies with reduced toxicity and improved long-term tolerability. In addition, the identification of predictive biomarkers that can stratify patients most likely to benefit from fucoidan-based combination therapy will be essential for the development of precision medicine approaches [14,27].

Recent advances in nanotechnology and drug delivery systems have further expanded the potential applications of fucoidan-based combination therapy. Because of its excellent biocompatibility, biodegradability, and affinity for various tumor-associated molecules, fucoidan has attracted increasing attention as both a therapeutic agent and a functional biomaterial for nanoparticles, liposomes, hydrogels, and other drug delivery platforms. These systems enable the co-delivery of fucoidan and chemotherapeutic drugs or natural bioactive compounds, thereby improving drug stability, tumor-specific accumulation, controlled release, and overall therapeutic efficacy while minimizing systemic toxicity. Such multifunctional delivery systems may further expand the clinical applicability of fucoidan-based combination strategies [27,32].

Overall, the successful clinical translation of fucoidan-based combination therapy will require standardized fucoidan preparations, comprehensive pharmacokinetic characterization, rigorous validation of synergistic interactions, and well-designed clinical studies. Another important goal is to identify optimal therapeutic partners, particularly natural bioactive compounds with complementary molecular mechanisms, to maximize therapeutic efficacy while minimizing toxicity. Overcoming these challenges will help advance mechanism-based, multitarget combination therapies and support the potential clinical application of fucoidan as an adjuvant for cancer treatment.

## 6. Conclusions

Fucoidan has emerged as a promising marine-derived polysaccharide for cancer combination therapy based largely on preclinical evidence demonstrating its multitarget biological activities, favorable safety profile, and ability to enhance the effects of diverse anticancer agents. In addition to its activity as an independent anticancer agent, in vitro and in vivo studies suggest that fucoidan may be particularly useful as a combination partner for conventional chemotherapy, targeted therapy, and, to a lesser extent, natural bioactive compounds. However, clinical evidence supporting the efficacy of these combination strategies remains limited.

Current preclinical evidence suggests that the potential benefits of fucoidan-based combination therapy result from the regulation of multiple molecular pathways, including apoptosis, autophagy, immune modulation, angiogenesis, and oncogenic signaling associated with therapeutic resistance. Although considerable progress has been made in combinations with conventional anticancer therapies, studies involving natural bioactive compounds remain limited. Further studies in this area may help develop safer, multitarget therapeutic strategies with reduced toxicity and improved therapeutic efficacy.

Future research should focus on standardizing fucoidan preparations, improving pharmacokinetic characterization, identifying optimal therapeutic partners and predictive biomarkers, and evaluating safety and efficacy in well-designed clinical studies. Based on current preclinical evidence, colorectal, breast, pancreatic, and hepatocellular cancers may warrant further investigation, although more clinical evidence is needed to determine which cancer types are most likely to benefit. Early-phase clinical trials using well-characterized fucoidan preparations will be important to establish appropriate doses and safety, while standardized manufacturing and quality control will also be necessary for future clinical and regulatory applications.

## Figures and Tables

**Table 1:** Representative studies investigating fucoidan-based combination therapy in cancer.

Combination Partner	Therapeutic Category	Cancer Model	Major Mechanism/Findings	Reference
5-Fluorouracil	Chemotherapy	Colorectal cancer (in vitro and in vivo)	↓ c-MET/KRAS/ERK, ↓ PI3K/AKT; ↑ chemosensitivity and apoptosis	Huang et al., 2021 [22]
Cisplatin/ Doxorubicin/Paclitaxel	Chemotherapy	Breast cancer (in vitro)	G2/M arrest, caspase activation; ↑ apoptosis; reduced toxicity toward non-malignant breast epithelial cells	Abudabbus et al., 2017 [21]
Cisplatin/Doxorubicin	Chemotherapy	Breast cancer (in vitro)	Autophagy activation (↑ LC3B, ↓ p62); ↑ chemosensitivity	Zhang et al., 2021 [37]
Oxaliplatin	Chemotherapy	Pancreatic cancer (in vitro and in vivo)	TLR4/NF-κB-mediated M1 macrophage polarization; tumor microenvironment remodeling	Deng et al., 2024 [24]
Sorafenib/Bevacizumab	Targeted therapy	Hepatocellular carcinoma (in vitro and in vivo)	↓ VEGF/PI3K/AKT/mTOR and KRAS/BRAF/MAPK signaling; ↑ caspase-dependent apoptosis	Abdollah et al., 2023 [23]
Lapatinib	Targeted therapy	HER2-amplified cancers (in vitro)	Cell type-dependent enhanced or reduced effects	Oh et al., 2014 [38]
Resveratrol	Natural bioactive compound	Colorectal cancer (in vitro)	Caspase activation and PARP cleavage; ↑ apoptosis	Vishchuk et al., 2013 [25]
Vitamin C	Natural bioactive compound	Colorectal cancer (in vitro and in vivo)	ROS-mediated apoptosis; ↑ cell death	Al Monla et al., 2022 [26]

Abbreviations: 5-FU, 5-fluorouracil; HER2, human epidermal growth factor receptor 2; MAPK, mitogen-activated protein kinase; NF-κB, nuclear factor kappa B; PARP, poly(ADP-ribose) polymerase; PI3K, phosphoinositide 3-kinase; ROS, reactive oxygen species; TLR4, Toll-like receptor 4; VEGF, vascular endothelial growth factor; ↑, increased; ↓, decreased.

## Data Availability

No new data were created or analyzed in this study. Data sharing is not applicable to this article.
